# Defective Nutrition in Early Life

**Published:** 1918-02

**Authors:** Elias H. Bartley

**Affiliations:** Brooklyn, N. Y.


### DEFECTIVE NUTRITION IN EARLY LIFE.

By Elias H. Bartley, M.D., Brooklyn, N. Y.

It has been estimated that approximately 10 per cent. of the children in the schools of our large cities suffer with malnutrition. There can be no doubt that a proper bodily nutrition is essential to bodily health and efficiency. It cannot be doubted that there are many persons in every community whose efficiency is impaired by malnutrition. This affects the efficiency of the community just in proportion to the number of such cases. It is then a matter of great importance to inquire into the causes and measures for the relief of these cases of malnutrition. Many, if not most cases, of undernutrition, except those developed by disease in later life ,originate in early life, i. e., before the sixth year. It is the cases of malnutrition which we meet during the first six to ten years of life that I shall have in mind in this discussion. The first question that presents itself is, to what extent is this condition hereditary or inborn and to what extent is it developed. Some cases are certainly hereditary, while others are as certainly developed by unfortunate surroundings, disease or accident. Among the diatheses so much discussed a few years ago, the asthenic ditheses was one.

By a diathesis was meant a condition of body or constitutional anomaly which predisposes to other pathological conditions. Asthenia universalis is a term applied by B. Stiller, of Budapest, in connection with certain individuals who have physically degenerated and show marked atonic condition with dyspepsia and neurastheni. He believes that some persons are born into this condition and remain in it with little chance of being lifted out of it. Acquired asthenia is the result of faulty hygiene and faulty metabolism, due to either faulty methods of feeding or the result of disease, and often dates back to the infancy or early childhood and by reason of inertia or neglect remain uncorrected as the years go by. It is evident that any reconstructive efforts to be successful must be begun in early life. When practicable such children should be transferred from city to country life, as a city life is too strenuous for them, and an outdoor life will do much to increase their general tonicity. Much more can be done for the acquired cases than for the hereditary cases, although, by careful management these latter may be greatly benefited, especially if they can be put under the care of a judicial nurse or governness. A great many neurotic mothers are not fit to raise their own children because they are too unstable to maintain a fixed policy and to manage a sensitive child.

Many of these underfed, mostly neurotic or neurashthenic children are injured during infancy or early childhood by faulty feeding, by acute infections such as the exanthemata, tonsilar infection or acute gastro-enteric infections.

These children are naturally regarded as delicate, therefore they are brought up in the idea that they are to be nursed, pampered and indulged.

Not rarely they come to us with a card from the school, as suffering with malnutrition, or, possibly from general tuberculosis. Much can be done for these cases of under nutrition and lowered vitality by careful management if efforts are begun early, or during the pre-school age, or before the effects of vicious habits are too firmly fixed. It is at this time that the remedial defects in children are often neglected or overlooked. During these years, when the general health and bodily characteristics are being formed, the responsibility for the future citizen rests upon the parents and the family physician. It is the up-to-date family physician, not the specialist, who can make good citizens of many of the cases we are discussing. He is in a position to follow up the supervision with the family, which is essential to obtain results.

By the time the child reaches the age of six or seven years, the chance of changing the general bodily health and constitution grows rapidly less.

The first necessity is to obtain a complete history of both child and parents; the home life, disposition, characteristics and habits. This takes time and patience, and will often inspire confidence and perhaps the co-operation of the parents without which we can do nothing. Much will depend also upon the practicability and the definiteness of directions and the ability and willingness of the parents to carry out the measures recommended.

These directions should include the regulations of the daily habits of exercise, rest, play, sleep, entertainment, diet, the care of the mouth, nose and throat. Since we cannot take time here to take up all these matters in detail, I want to say a few words about some of them.

These children are usually treated as if they were invalids, and are often pampered too much. This is a mistake. While we cannot always practice the so-called hardening process- by exposure to extreme changes of temperature and weather, we can allow them as much liberty of action as their health will permit, and much more than parents are inclined to do. Fresh air at night is now easily obtained, thanks to the influence of the fresh air propaganda. All children should be made to walk as soon as they can do so redily, instead of being wheeled about in a go-cart. They should not be hemmed about with too many don'ts. Give them range enough to let them develop, with just enough oversight to protect them from bodily harm. It is sometimes surprising to see the improvement in an apparent weakling who has been restrained at every turn from this kind of liberty.

It goes without saying that long hours of sleep, a proper amount of rest after the tire of play or walking must be enjoined. This should be systematic and not haphazard. Entertainment—so-called—is often carried to the ridiculous. To allow a child of five or six years to attend the theatre or picture show, especially those of the type of children we are considering, is a great mistake. It excites and exhausts the nervous system to an alarming degree.

As the mouth is the port of entry of all that nourishes the body, so also is it the port of entry of infections that determine the health or ill health of the body. It is only in recent years that we have come to realize the great influence of oral, nasal and tonsilar infections on general health. Dental malocclusion, dental caries, pyorrhoea, adenoid growths, sinus disease, mouth deformities, tonsilar infections, all have a very important effect on the development of children, as well as their general health. It is now well known that many of our diseases originate in these infections; as for example, rheumatism, so-called, either acute or chronic deforming, endocarditis, gastric disorders, including gastric and duodenal ulcers, appendicitis and gall bladder infections, deafness, mastoiditis, some cases of pneumonia, and possibly others.

It has been observed by some authors that tubercular children ususlly have bad teeth. Dr. Knepf says, "I defy the most skilled physician to either cure or help a tubercular patient who has decayed teeth in his mouth. The restoration of the teeth increases greatly the chances of recovery."

On the management of these physically defective children, wo should carefully inspect and treat, or have corrected, any source of infection in the mouth, nose and throat.

In recent years, especially in large cities and suburbs, infections have grown more frequent and in some cases more virulent, and hence the secondary diseases such as cardiac diseases, renal diseases, pneumonia, mastoiditis, gastric and duodenal ulcers, are apparently on the increase. As the people in the country living near large cities more frequently visit the city now than before the advent of the automobile, these remarks apply to the country as much as to the city. The question of nutritional disorders in childhood is a common one and a difficult one to handle.

The chief difficulties we have to contend with are:

1. The prejudices and preconceived notions of the mother or nurse.
2. The previous habits and prejudices of the child.
3. The lack of appetite or digestion of the child.
4. The unwillingness of the child to assist.

**The Notions of the Mother or Nurse.**—Frequently the most difficult mother to manage is the one who reads a popular book, or some other authority on foods. I was recently called to treat a child of twelve months for malnutrition. It was very anemic, had no teeth, could not walk, and had been losing weight for four months. I found that the mother had read that green vegetables contained iron. She was giving him spinach, celery and lettuce every day and still he lost weight. By a sensible change in diet, omitting these "greens," he gained color and five pounds in three weeks and began to walk.

Another preconceived notion of mothers is, that a child should be made to eat the things she thinks are good for him, or go without; the result being that the child expects a contest every time he comes to the table. This method of trying to force a child to eat seldom succeeds in securing the object desired. This notion of mothers must be corrected to one of coaxing the child's appetite rather than driving it. This can usually be done by catering to the child's likes and dislikes so far as substantials go. Find out by numerous trials, what apepals to the child, by ever changing variety, avoiding nick-knacks which benumb the appetite.

Most mothers think that a child of two years must eat soft boiled eggs and meat juice to live. They do not dare to allow a fried egg, or an omelet, or fried potatoes or fried mush. He must have a chop or roast meat, never salt meat. If a child of two to four years take milk he does not need egg or meat of any kind. He gets all the potein he needs in the form of caein and albumin and in a better form than as meat. It is a very common observation that the child who drinks milk freely is stronger, grows to a larger stature than other children of the same family who do not drink milk. I regard it a misfortune when a child does not take milk. Rarely we meet with cases of milk anaphalaxis, but more often we find that the mother is to blame for the distate for milk. At the weaning period she allows other foods to displace milk, or, because she does not like milk she causes her child to dislike it because of digestion. This I have often observed. Efforts should be made in such cases to induce the child to return to milk in some form, as junket, custards, malted milk, etc. When possible a lacto-farinaceous diet will generally be found to give the best results. Vegetables will be a useful addition to these, letting the child at first select the vegetables it is willing to eat. Among the farinaceous foods, allow the greatest possible liberty of choice and method of cooking. Cast theory to the dogs, when necessary, in methods of cooking or selection of cereal. The main object with these children is to promote appetite and nutrition. When milk cannot be used, for any of the above reasons, the difficulty is greater. Meat, cheese, legumes or eggs will be needed. If fresh meats are distasteful, it is usually because of monotony in the selections. It must be known that salt meats are often more palatable and quite as digestible as fresh meats. Cold corned beef, tongue, bologna, dried beef (smoked beef), ham and bacon are usually well borne in reasonable amount by children three or four years old. Those meats which have been smoked in brine as a part of the curing process, are especially desirable because the connective tissue has been disintegrated so that in mastication the tendency is to crumble and expose a greater surface to the digestive juices than is the case with fresh meats. It is a law of chemical action that the rapidity of reactin is proportionate to the surface exposed. It is for this reason that bread is raised, and that bread that is old eonugh to be brittle is more easily and rapidly digested than new bread which is doughy and plastic. For the same reason hot biscuits, griddle cakes,, dumplings, sweet potatoes and bananas are less easily digested than some other foods. The modern steamed oats, for the same reason, is less easily managed than the old-fashioned cracked oats, and the latter should be selected. However, some of these foods may be allowed if on trial they appeal to the appetite and are well borne. I would expect the bananas for other reasons.

**Fats.**—Most of these children discard fats as much as much as possible and no efforts are usually made to correct this vice. The cells of the body all contain from 15 to 25 per cent. of fat as an essential part of their composition, and hence it is essential to the growth and maintenance of the life of these cells. To be sure when fats are not supplied carbohydrates, proteins and the tissues themselves can be and are made to furnish fats. But this contains considerable loss of energy, and the body prefers to receive the fat as such. This is shown by the fact that when it is supplied freely the body stores it up without change. Thus if a dog is fed goose fat freely, analysis shows that the fat stored up is not all dog fat but goose fat. If the dog is starved the goose fat is used up just as reaily as the dog fat. Fat is essential to the well being of all growing children. It can most readily be supplied by milk, butter, bacon and gravies, all readily taken by most children and easily digested. Even when milk is not well borne, butter is usually well tolerated during the second year and later, and I have come to regard it as a great assistance in the treatment of all cases of malnutrition. For many years I have made it a practice to ask consumptives if they like fats. In the vast majority of cases a look of disgust is the answer. Sometimes the abstinence from fats is an early symptom of tuberculosis of the lungs, and I have thought at times this contributes largely to the progress of the disease.

At any rate the free use of fat may retard its progress to a marked degree. For this reason I regard bacon and butter in liberal allowance as highly beneficial in all cases of malnutrition whether due to tuberculosis or not. The use of cod liver oil in tuberculosis is too well known to need discussion. The storage of fat in the tissues adds to the nutrition of the cells, gives added strength, protects the body from loss of heat by radiation, gives rotoundity to the figure and serves as fuel for use during temporary cessation of food in illness to which all of us are liable.

**Sugar.**—Sugar is the most easily metabolized of the food constituents, especially malt sugar. The only drawback is that when used too freely it has certain disturbing effects on the digestion of other necessary foods. It benumbs the appetite especially when taken between meals or on an empty stomach. Therefore, it should be eaten with the meals unless it is found to produce acid fermentation. These undernourished children are apt to be very fond of everything sweet, and many of them will not eat anything else if allowed to indulge this tendency. The tolerance of sugar must be studied in each case. When well tolerated and when taken with meals it acts to protect the proteins and fats to a large degree and it promotes the retention of water which is desirable. The too free use of sugar tends to increase acidemia, which is a frequent tendency in such children.

This brings me to speak of another matter of considerable importance, and one frequently overlooked. This is the balance in acid-forming and alkali-forming foods. A dietary should be such that there is a slight excess of alkaline residue left after complete combustion, to maintain the proper degree of alkalinity of the blood and lymph. Otherwise the child suffers with acidemia or a sub-alkalinity of these tissues, and may develop scurvy.

In general the meats, eggs, cereals, including rice, are acid-forming foods.

The fruits, green vegetables, milk, potatoes, legumes and milk are alkali forming foods. Meat soups, meat juice and cereals without milk or vegetables, then, must be regarded as an improper continuous diet. When they are used their acid residue must be neutralized by potatoes, vegetables, fruits or milk. The so-called anti-scorbutic foods, it will be observed, are all alkali-forming foods, and there are good reasons to believe that scurvy may be induced by this form of acidemia independent of the absecene of the socalled vitamines. This is one very important element in the failure to grow and develop in some of these cases of malnutrition.

The reason why orange or other fruit juices act so promptly in the cure of scorbutis is that they supply inorganic bases to alkalize the tissues. Children are more likely to suffer from acidemia than adults, because of their more restricted diet.

Experiments on mice show that they die in eleven to fifteen days, when fed on an ash free diet of pure casein, fat and sugar, and do not better when sodium chloride is added to this diet. They live twice as long when sodium carbonate is added. Taylor developed marked symptoms on practically an ash free diet. Goode and Joslin tried the same experiment but did not develop these symptoms. Wright, who studied the epidemic of scurvy during the siege of Ladysmith, says the disease followed a diminished alkalinity of the blood due to food which furnish too little of the bases. Gautier states that the outbreak of scurvy during the siege of Paris was not connected with the use of salt meat, but with the exhaustion of the supply of vegetables. The diet of children then should be so chosen as to furnish the body with enough base-forming elements to neutralize the acids produced from the metabolism of the sulphur produced from the metabolism of the sulphur and phosphorus of the food eaten. This is a strong argument against the too free use of soups in the diet of young children.

**Discussion.**

Dr. A. Clifford Mercer, Syracuse: Every physician present would agree, I think, with Dr. Bartley when he says:

"The question of nutritional disorders in childhood is a common one and a difficult one to handle."

I believe, farther, that no two physicians would handle this common and difficult problem in a given case in the same way, and that no one physician would attempt to solve the problem in just the same way in two different cases. The factors used in the solution of the problem are at least more numerous than the letters of the alphabet from which are formed all the words and sentences of the English and numerous other languages. So, therefore, the number of possible combinations of these factors is greater than the number of words in an unabridged Webster, and the actual combinations are probably as numerous as the patients of all the physicians who seriously deal with the nutritional disorders of the first six years of life, which period Dr. Bartley says he has in mind in his discussion.

Even during the first year of this period, when conditions may be pretty well controlled, no hard and fast rules can be laid down, while with the passing years conditions become more complex and less subject to control and, therefore, less subject to hard and fast rules.

Dr. Bartley's paper is full of suggestive details, too numerous for one in the allotted time to discuss in turn even briefly.

Speaking from my own point of view I am led to believe that the most universal fault in the majority of cases in question is neglect of correct habits in relation to the passage of food stuffs or their derivatives through the beginning and end of the alimentary canal. In infancy it is the irregular hours of feeling, following the whims of the baby or convenience of the mother, with food perhaps right in quality and quantity, but more commonly wrong in these respects. In older children it is the eating between meals, spoiling the appetite for meals, and the neglect of a regular stool habit. Even with good food, the results may be disastrous, but with food wrong in character and amount the results are obvious, although it may be secured with corresponding worse. The treatment indicated difficulty.

In regard to the character of the food given to older children my usual practice is to advise mothers to use as a guide the food lists found in Dr. Holt's little book, "The Care and Feeding of Children." I tell them when a neighbor or relative suggests a food the child has not had before to consult the book and give the food only if it is in the approved list, and certainly not if it is in the forbidden list. If in doubt about any point I ask the mother to talk the matter over with me. Mothers who are capable soon learn to make good use of this guide, and to some the little book becomes almost a second Bible. Its teachings become in a very satisfactory way general fundamental subjects for discussion which are suppplemented by modifying or additional advice to meet particular conditions.

Finally, I would add that when we take credit unto ourselves for a successful result it is at least wise to take into account the elasticity and adaptability of the digestive function. Depending upon this adaptability I feel reasonably sure that many of us consciously or unconsciously often really train a baby to thrive on the food which we for theoretcial and clinical reasons see fit to prescribe. Perhaps, for instance, the following teaching of an enthusiast, one trains the infant to take a proportionately excessive percentage of fat, and successfully up to a certain point, but only more intelligently than the Italian tenement mother in a negative way trains her baby to take during its first year substantial adult food with remarkable impunity.—New York State Journal of Medicine.

